# Pyothorax Caused by Arcanobacterium haemolyticum and Staphylococcus aureus Co-infection: A Case Report

**DOI:** 10.7759/cureus.44096

**Published:** 2023-08-25

**Authors:** Yoriko Herai, Takumi Nakahara, Kouji Kasai, Shohei Omori, Keiichi Tokuhiro

**Affiliations:** 1 Infectious Diseases, Chiba University Hospital, Chiba, JPN; 2 Respiratory Medicine, Misato Central General Hospital, Misato, JPN; 3 Internal Medicine, Misato Central General Hospital, Misato, JPN; 4 Laboratory of Microbiology, Ageo Medical Laboratory, Ageo, JPN; 5 Orthopedic Surgery, Misato Central General Hospital, Misato, JPN; 6 Cardiovascular Surgery, Misato Central General Hospital, Misato, JPN

**Keywords:** diabetes mellitus, co-infection, pyothorax, staphylococcus aureus, arcanobacterium haemolyticum

## Abstract

*Arcanobacterium haemolyticum *causes pharyngeal and skin lesions but rarely causes severe systemic infections. An 80-year-old woman with diabetes mellitus was admitted for surgery of a left femoral neck fracture and right first toe ulcer. On day 19, chest radiography revealed a massive left pleural effusion.Pleural fluid culture grew *Staphylococcus aureus* and* A. haemolyticum*. The fluid was drained via a chest tube, and the patient was treated with cefazolin and clindamycin. Only four cases of pyothorax caused by *A. haemolyticum* have been reported, and no previous cases of *A. haemolyticum* pyothorax with bacterial co-infections have been reported.

## Introduction

*Arcanobacterium haemolyticum* was first isolated as *Corynebacterium haemolyticum* from pharyngitis and skin lesions by MacLean et al. in 1946 [1]. In 1982, it was transferred from *Corynebacterium haemolyticum* to* A. haemolyticum *due to deficiencies in chemical and numerical phenetic data [2]. *A. haemolyticum* is a gram-positive-to-variable rod, catalase-negative, facultatively anaerobic, non-motile, non-spore-forming, and variably β-hemolytic on sheep blood agar. Matrix-assisted laser desorption ionization time-of-flight mass spectrometry is useful for rapid and accurate identification [3].

*A. haemolyticum* is an obligate parasite of the human pharynx that causes sporadic pharyngeal or skin lesions [3]. Rarely, *A. haemolyticum* can be associated with severe systemic infections, such as infective endocarditis [4,5], abscesses [6-8], and sepsis [9]. *A. haemolyticum* is usually the sole isolate present in blood cultures. In contrast, it is usually the predominant isolate in polymicrobial populations in cultures from nonsterile sites [10]. To our knowledge, only four cases of pyothorax caused by *A. haemolyticum* have been reported [9-12], and no previous cases of *A. haemolyticum* with bacterial co-infections have been reported. We report a case of pyothorax caused by co-infection with *A. haemolyticum* and *Staphylococcus aureus*.

## Case presentation

The patient was an 80-year-old Japanese woman with a history of cerebral infarction at the age of 58 years and Alzheimer's disease at the age of 63 years. She had memory deficits (inability to recognize family members), speech limited to fewer than five words, total functional dependence, and incontinence and was unable to walk, so received care in long-term facilities.

She was admitted for surgery of a left femoral neck fracture and right first toe ulcer. The ulcer in her right first toe was likely to have also been complicated by osteomyelitis. The tests performed at the time of admission revealed the presence of diabetes mellitus.

**Figure 1:**
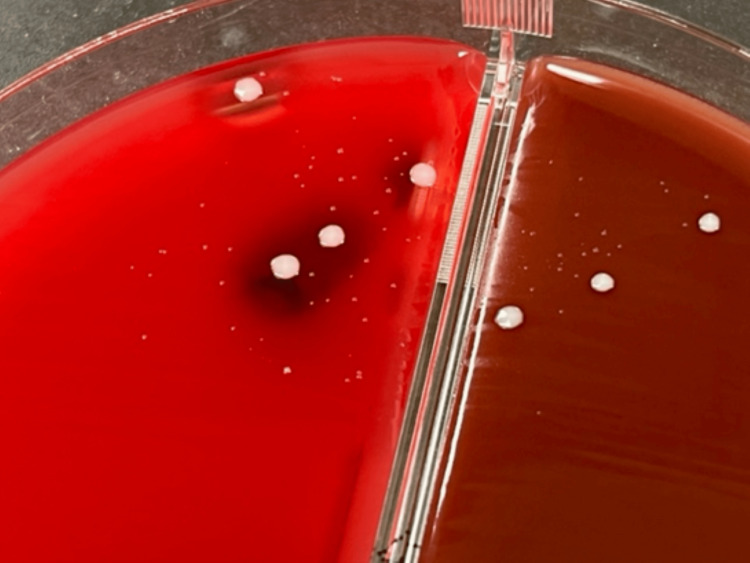
Aerobic culture on 5% sheep blood agar after 24 hours at 37°C showing minute, large, and small colonies.

On day 7 after admission, she underwent a left above-knee amputation of the left leg as the left leg was highly deformed due to fractures and contractures and was at high risk of rubbing against the right leg and forming an ulcer. On day 9, she was scheduled to undergo amputation of the right thumb, but she developed fever and was diagnosed with a urinary tract infection, which was treated with ceftriaxone intravenous 2g injection (IV) every 24 hours. On day 11, the antibiotic was changed to ceftazidime 1g IV every eight hours due to poor effectiveness and *Pseudomonas aeruginosa* was detected in the urine culture. On day 19, chest radiography revealed a massive left pleural effusion even though there were no abnormalities previously (Figure 1). There were no abnormal findings in the pharynx or signs of infection in the surgical wound in the left inguinal area. The right first toe ulcer remained unchanged since admission.

**Figure 2:**
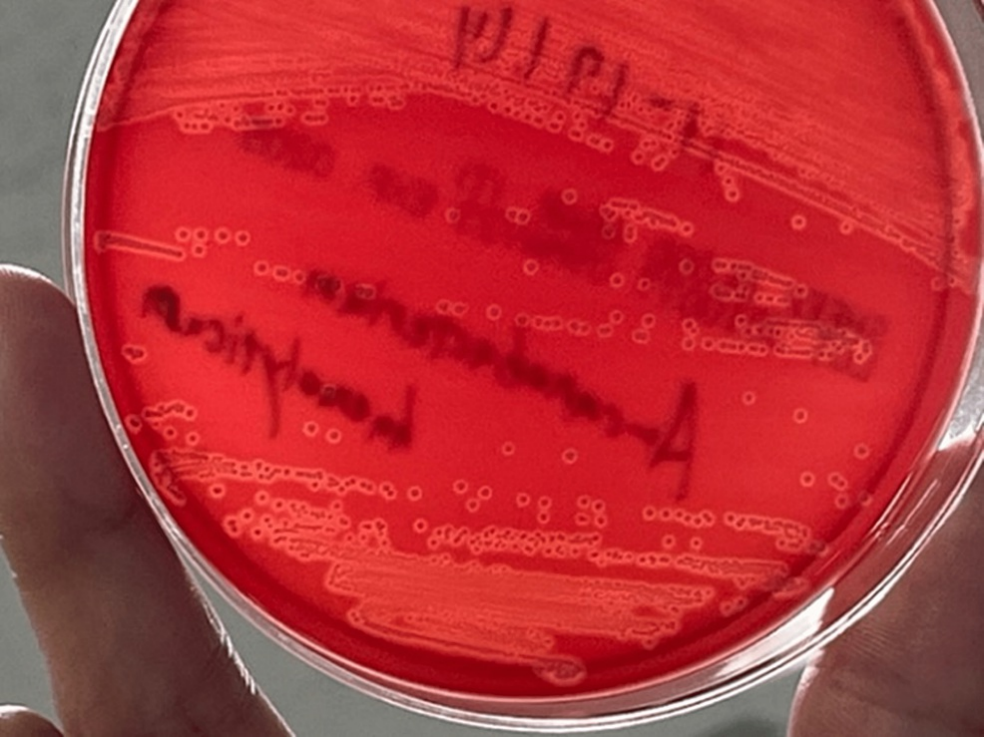
Aerobic culture on 5% sheep blood agar after 24 hours at 37°C showing minute, large, and small colonies.

**Figure 3:**
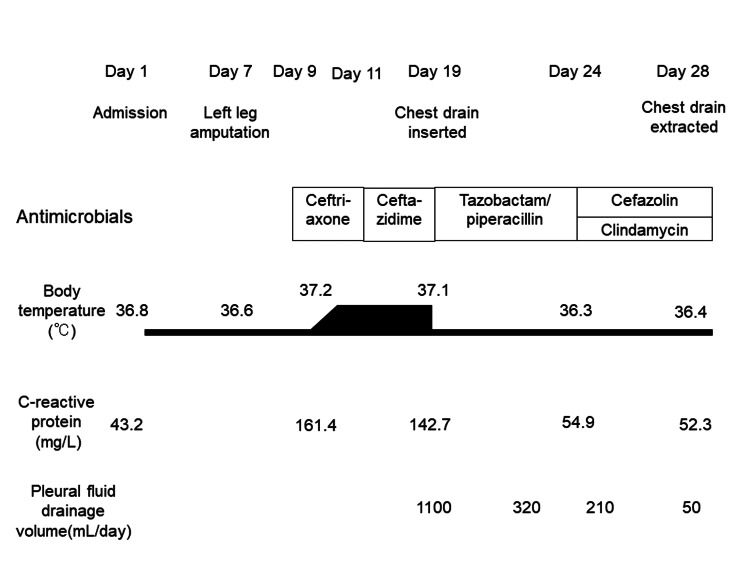
The patient’s clinical course.

The pleural fluid was purulent, and biochemical tests, cell counts, and cell differential showed an exudative pleural, lactate dehydrogenase (LDH) 5473 U/l, total protein (TP) 30 g/l, adenosine deaminase (ADA) 84.7U/l, white blood cell count 11453/µL of which neutrophils were 93.2%. The gram stain of the pleural fluid showed Gram-positive cocci. Blood biochemistry revealed a positive inflammatory reaction, hypoalbuminemia, and hyperglycemia. The patient was diagnosed with a pyothorax and underwent chest tube drainage. Microscopy of a Gram-stained smear of the pleural effusion showed Gram-positive rods. The pleural fluid culture at the time of thoracic drainage grew two colonies on 5% sheep blood agar after 24 hours at 37°C (Figure 2). The larger colony contained Gram-positive cocci, and the smaller colony contained Gram-positive rods. After 48 hours of incubation on 5% sheep blood agar, the small colonies showed hemolysis (Figure 3). These bacteria were identified as *S. aureus* and *A. haemolyticum* using an automated identification test (MALDI Biotyper, Bruker Daltonik GmbH, Bremen, Germany). We attempted to determine antimicrobial susceptibility using the microdilution method, but *A. haemolyticum* did not develop.

On day 19, the antimicrobial was changed to tazobactam-piperacillin 4.5g IV every eight hours due to assumed anaerobic bacterial infection. The patient began to require frequent expectoration of sputum and developed recurrent aspiration pneumonia. On day 24, the antimicrobial to cefazolin 2g IV every eight hours and clindamycin 600mg IV every six hours based on the culture testing results. On day 28, the patient’s pleural effusion decreased, and the chest drain was removed (Figure 3). There was no recurrence of pyothorax after drain removal. 

## Discussion

*A. haemolyticum* causes pharyngitis and skin and soft tissue infections [3].* A. haemolyticum* can also cause systemic infections, such as Lemierre syndrome [13], brain abscess [14-16], infective endocarditis [4,5], abscesses of the pelvis [8], thyroid gland [7], or liver [6], and spontaneous bacterial peritonitis [17].

We conducted a PubMed search for case reports and case series on *A. haemolyticum* pyothorax published in English up to March 25, 2023. The search terms used were “[Arcanobacterium haemolyticum] OR [Corynebacterium haemolyticum].” The literature search revealed four published case reports (Table 1) [9-12].

**Table 1:** Characteristics of five cases of pyothorax due to Arcanobacterium haemolyticum.

Case no.	Ref. no.	Age (years)	Sex	Underlying conditions	Entry site of infection	Co-infection	Antibiotic therapy	Outcome
1	Present case	85	Female	Diabetes mellitus	Foot ulcer	Staphylococcus aureus	Cefazolin and clindamycin	Recovery
2	[10]	20	Male	None	Pharyngitis	Mycoplasma pneumoniae	Not noted	Recovery
3	[11]	19	Male	None	Unknown	None	Ceftriaxone and metronidazole	Recovery
4	[9]	26	Male	Epilepsy	Pharyngitis	None	Ampicillin/sulbactam	Recovery
5	[12]	20	Male	None	Pharyngitis	None	Vancomycin and piperacillin/tazobactam	Recovery

*A. haemolyticum* is usually the sole isolate present when isolated from blood cultures. In contrast, it is usually present as the predominant isolate in a polymicrobial population in cultures from nonsterile sites [18]. A literature review revealed several cases of co-infection at sterile sites [15]. However, we did not identify any previous reports of bacterial co-infection in cases of pyothorax. In this case, *A. haemolyticum* and *S. aureus *were detected, but neither was predominant. In cases where co-infection is found at sterile sites, this could reflect infection at the entry site.

Diabetes mellitus and malignancy are underlying conditions associated with severe *A. haemolyticum* infection. Conversely, sepsis due to* A. haemolyticum* can occur even in the absence of comorbidities. Our patient had diabetes mellitus, but no immunosuppressive comorbidities were reported in the other four cases of *A. haemolyticum* pyothorax identified in the literature search (Table 1).

A previous study tested the susceptibilities of 138 clinical isolates of *A. haemolyticum* to 11 antimicrobial agents [19]. All strains were susceptible to phenoxymethylpenicillin, cephalosporins, erythromycin, azithromycin, clindamycin, vancomycin, doxycycline, and ciprofloxacin but were resistant to trimethoprim-sulfamethoxazole. We did not obtain antimicrobial susceptibility results in our patient.

Even with systemic infections, the prognosis is good, and no cases were found to have a direct cause of death. In previous reports, only one death was reported which was due to cerebrovascular disease and not due to *A. haemolyticum* infection [20].

## Conclusions

Pyothorax caused by A. haemolyticum is very rare, and this is the first case of pyothorax with co-infection to be reported. We report this case because knowledge regarding A. haemolyticum pyothorax, including antimicrobial susceptibility, is limited, and owing to the rarity of this condition, it is important to accumulate more cases to improve knowledge.
